# *Acinetobacter baumannii* Outer Membrane Protein A Induces Pulmonary Epithelial Barrier Dysfunction and Bacterial Translocation Through The TLR2/IQGAP1 Axis

**DOI:** 10.3389/fimmu.2022.927955

**Published:** 2022-06-30

**Authors:** Wang Zhang, Hua Zhou, Yan Jiang, Jintao He, Yue Yao, Jianfeng Wang, Xiaochen Liu, Sebastian Leptihn, Xiaoting Hua, Yunsong Yu

**Affiliations:** ^1^ Department of Infectious Diseases, Sir Run Run Shaw Hospital, Zhejiang University School of Medicine, Hangzhou, China; ^2^ Regional Medical Center for National Institute of Respiratory Diseases, Sir Run Run Shaw Hospital, Zhejiang University School of Medicine, Hangzhou, China; ^3^ Key Laboratory of Microbial Technology and Bioinformatics of Zhejiang Province, Hangzhou, China; ^4^ Department of Respiratory and Critical Care Medicine, The First Affiliated Hospital, Zhejiang University School of Medicine, Hangzhou, China; ^5^ Department of Respiratory and Critical Care Medicine, Zhejiang Provincial Hospital of Chinese Medicine, Hangzhou, China; ^6^ Zhejiang University-University of Edinburgh Institute, Zhejiang University, Haining, China; ^7^ University of Edinburgh Medical School, Biomedical Sciences, College of Medicine and Veterinary Medicine, The University of Edinburgh, Edinburgh, United Kingdom

**Keywords:** *Acinetobacter baumannii*, outer membrane protein A, epithelial barrier, bacterial translocation, cytoskeleton

## Abstract

Pulmonary epithelial barrier dysfunction is a critical pathophysiological process in pneumonia and associated invasive infections, such as those caused by *Acinetobacter baumannii*. However, the mechanisms underlying *A. baumannii*-induced pulmonary epithelial barrier dysfunction and bacterial translocation remain unclear. In this study, lungs of mice and A549 human epithelial cell monolayers were challenged with the *A. baumannii* wild-type strain and an outer membrane protein A (*ompA*) deletion strain. In addition, epithelial cells in culture were treated with purified OmpA protein or transfected with a eukaryotic expression vector encoding *ompA* (pCMV-*ompA*). Bacterial translocation across cell monolayers and intrapulmonary burden were measured, barrier function was evaluated *in vivo* and *in vitro*; cell migration ability was determined. The specific inhibitors C29 and JSH-23 were used to suppress the activity of Toll-like receptor 2 (TLR2) and of NF-κB, respectively. IQ-GTPase-activating protein 1 (IQGAP1) small interfering RNA was used to knock down endogenous IQGAP1 expression. In this work, we show that OmpA from *A. baumannii* increased the production of pro-inflammatory cytokines, remodeled the cytoskeleton, and internalized intercellular adherens junctions (AJs); these changes eventually induced pulmonary epithelial barrier dysfunction to promote bacterial translocation. IQGAP1-targeting small interfering RNA and chemical inhibition of TLR2 or NF-κB prevented high permeability of the pulmonary epithelial barrier. TLR2/NF-κB signaling was involved in OmpA-induced inflammation, IQGAP1-mediated OmpA-induced opening of the pulmonary epithelial barrier *via* cytoskeleton dynamic remodeling, and cellular redistribution of the major AJ protein, E-cadherin. These observations indicate that *A. baumannii* uses OmpA to overcome epithelial defences and cross the pulmonary epithelial barrier.

## Introduction

As an opportunistic pathogen, *Acinetobacter baumannii* has been identified as pathogen of critical priority by the World Health Organisation and included in the “Global priority list of antibiotic resistant bacteria to guide research, discovery and development of new antibiotics, 2017” (1). *A. baumannii* is mainly prevalent in hospital environments, particularly in intensive care units where it often is observed in pneumonia and bacteraemia patients (2). Compared to patients with ventilator-associated pneumonia alone, the mortality rate of ventilator-associated pneumonia patients with secondary *A. baumannii* bacteraemia is greater than 30% (3). Therefore, *A. baumannii* infection is a major challenge worldwide, effective treatment strategies for multidrug resistant *A. baumannii* infections are not available.

The airway epithelium is the first line of defence against invasion by pathogenic microorganisms. The epithelium continuously and dynamically regulates its barrier function in response to environmental stimuli (4). However, certain pathogen including *A. baumannii* have the ability to destroy the integrity of the alveolar epithelial barrier, resulting in acute lung epithelial injury and the dissemination of bacteria into the bloodstream (5). Comprehension of the mechanism of lung tissue destruction and invasion by *A. baumannii* is important to possibly prevent subsequent septicaemia. The outer membrane protein A (OmpA) of *A. baumannii* is a key virulence protein associated with persistent pulmonary infection. Previous analysis based on clinical samples showed that the expression level of OmpA was positively correlated with the severity of the disease and the risk of death (6). *In vivo* experiments showed that the presence of OmpA increased the bacterial load in the blood, while inducing serious lung pathological changes, in infected mice (7). *A. baumannii* OmpA invades epithelial cells through a zipper-like mechanism by affecting microtubule and microfilament condensation (8), suggesting that OmpA may affect cell function *via* targeted regulation of the cytoskeleton. Proteomic studies have confirmed that OmpA is a crucial protein in the *A. baumannii*–host interaction network (9); therefore, OmpA is presumably responsible for airway damage by *A. baumannii*. However, the specific mechanism of OmpA-mediated airway epithelial barrier dysfunction is unclear.

Toll-like receptor 2 (TLR2) is a pattern recognition receptor that mediates cellular activation *via* distinct microbial components (e.g., outer membrane vesicles derived from *A. baumannii*) and modulates pulmonary inflammation (10). TLR2 promotes bacterial clearance during the initial stage of pulmonary infection by *A. baumannii (* 11). There is increasing evidence to support a role for OmpA in the activation of TLR2, as well as an interaction between OmpA and TLR2 (12). IQ-GTPase-activating protein 1 (IQGAP1) is a typical host cytoskeleton protein that is involved in cell migration and the maintenance of cell–cell connections (13, 14). Recent evidence has suggested that IQGAP1 plays an important role in the invasion of host cells by pathogenic microorganisms (15, 16). The OmpA of *Escherichia coli* K1 interacts with endothelial cell glycoprotein 96 in the blood–brain barrier to enter the central nervous system; IQGAP1 mediates the disruption of cell–cell junctions induced by OmpA (17). IQGAP1 also influences actin polymerisation to regulate Salmonella invasion (18). Therefore, IQGAP1 has a core role in bacterial pathogenicity as a key regulator of actin cytoskeleton dynamics. In addition, IQGAPs have been shown to choreograph cellular signaling from the membrane to the nucleus, mediating many pathophysiological processes (e.g., inflammatory regulations); IQGAP1 knockdown significantly affected many cytokines and inflammatory factors with vital roles in acute lung injury and acute respiratory distress syndrome (19). These results suggest that IQGAP1 may have multiple roles in the pathogenesis of *A. baumannii* infection.

This study investigated whether *A. baumannii* OmpA induces pulmonary epithelial barrier dysfunction and bacterial translocation; and aimed to elucidate whether OmpA injured epithelial cells through the IQGAP1 complex and TLR2 signaling pathways.

## Materials and Methods

### Bacterial Strains

The WT *A. baumannii* strains ATCC17978 and ATCC19606 were used in this study (**Supplementary Table 1**). One-step chromosomal gene inactivation (20) based on recombination-mediated genetic engineering was used to establish *ompA* deletion strains (Δ*ompA*); the pYMAb2-*ompA* shuttle vector was used to establish *ompA*-complemented Δ*ompA ompA* ^+^ strains (17978Δ*ompA*/pYMAb2-*ompA*). WT and Δ*ompA A. baumannii* strains were grown in Mueller-Hinton Broth (Oxoid; Thermo Scientific, Waltham, MA, USA); Δ*ompA ompA* ^+^ strains were grown in Mueller-Hinton Broth supplemented with hygromycin (100 μg/mL). All strains were grown at 37°C with shaking for 12–16 h.

### Cloning, Expression, and Recombinant OmpA Protein Purification

The full-length coding sequence of *A. baumannii* ATCC17978 *ompA* (A1S_2840) was amplified using DNA from *A. baumannii* ATCC17978, then cloned into the pYMAb2 and pCMV-Flag vectors to generate pYMAb2-*ompA*, pCMV-*ompA* (unfused protein), and pCMV-Flag-*ompA* (fused protein). The full-length human *tlr2* sequence (NM_001318787) was amplified using cDNA prepared from A549 cells, then cloned into the pEGFP-N1 vector to generate pEGFP-*tlr2*. All plasmids were verified by DNA sequencing and are listed in **Supplementary Table 2**.

The bacterial expression and purification of His-tagged OmpA were performed as previously described (21) Briefly, the sequence encoding *ompA* (A1S_2840) without signal peptide was amplified using DNA from *A. baumannii* ATCC17978 and cloned into the pET28A vector to generate the pET28A-*ompA* construct. The confirmed pET28A-*ompA* construct was transformed into competent BL21 (DE3) cells and grown in Luria–Bertani (LB) broth with shaking at 200 rpm until the OD_600_ of bacteria culture was 0.8. Then isopropyl-β-d-thiogalactoside (IPTG) was added to a final concentration of 1mM to induce target protein production. Bacteria were collected and resuspended in lysis buffer. Then disrupted by JN-02C French Press (JNBIO, Guangzhou, China) at 1100 bar, supernatant was filtered through a pre-equilibrated Ni-column, proteins were washed and eluted with the elution buffer (lysis buffer with 300 mM imidazole). Eluate was further subjected to purification using a pre-equilibrated Superdex 75 column. The purified proteins were loaded in SDS-PAGE to assess the purity and treated to remove endotoxin before use.

### Cell Culture, Transfection, and Bacterial Infection

The A549 human pulmonary adenocarcinoma cell line (#CCL185; ATCC, Manassas, VA, USA), HBE human bronchial epithelial cell line (#CRL2741; ATCC), and HEK293 cell line (#CRL1573; ATCC) were cultivated in Dulbecco’s Modified Eagle’s medium (DMEM, Cat. No: 10-013-CV; Corning Inc., Corning, NY, USA) supplemented with 10% foetal bovine serum (FBS, 35-081-CV; Corning) and 1% penicillin/streptomycin (Cat. No: 30-002-CI; Corning) and incubated at 37°C in an atmosphere of 5% CO_2_. The TLR2 inhibitor C29 (Cat. No: HY-100461; MCE, Monmouth Junction, NJ, USA) and NF-κB inhibitor JSH-23 (Cat. No: HY-13982; MCE) were added to DMEM without FBS at concentrations of 50 μmol/L and 20 μmol/L, Small interfering RNA targeting IQGAP1 was transfected into A549 cells at 30 pmol per 1.0 × 10^5^ cells. Eukaryotic expression vectors were transfected into cells using Lipofectamine 3000 transfection reagent, in accordance with the manufacturer’s protocol (Thermo Scientific). For cell infection, FBS-free medium without penicillin/streptomycin containing bacteria at a multiplicity of infection (MOI) of 50 was added into cell plates and incubated at 37°C in an atmosphere of 5% CO_2_.

### Mouse Infection Models

Male C57BL/6J mice aged 7–8 weeks (WT) with body weight of 20–25 g were obtained from Hangzhou Ziyuan Laboratory Animal Technology Co., Ltd. (Hangzhou, China). Animals housed in a pathogen-free facility at a controlled temperature of 20–26°C, with humidity of 40–60% and free to access to food and water, were used to establish a pneumonia infection model that recapitulates hospital-acquired or ventilator-associated pneumonia. In brief, mice were anesthetized by the intraperitoneal injection of pentobarbital sodium (1%; 50 mg/kg), the tongues were held to expose glottis, 24G intravenous indwelling needle was used as the tracheal intubation to insert the airway, after confirming the successful insertion of the trachea, the needle core was pulled out and 50 μL bacterial (5 × 10^8^CFU/mL) suspension was injected into the trachea to allow the bacteria into the lungs by reflexive aspiration.

All animal work was conducted following approval by the Animal Care and Use Committee at the Sir Run Run Shaw Hospital, College of Medicine, Zhejiang University, and in compliance with the Guide for the Care and Use of Laboratory Animals published by the US National Institutes of Health (NIH Publication no.85−23; revised 1996).

### Histological and Immunohistochemical Analysis

Mice were anaesthetised and sacrificed by cervical dislocation, then subjected to whole-body perfusion. The tissues were fixed in 4% paraformaldehyde and cut into 4-μm-thick sections, which were then deparaffinised with xylene and rehydrated by passage through an alcohol series. Lung sections were stained with haematoxylin and eosin, then observed under a light microscope (Olympus, Tokyo, Japan). Immunohistochemical analysis was performed using a as previously reported protocol, as described in the **Supplementary Methods**.

### Bronchoalveolar Lavage and Evans Blue (EB) Dye Leakage *In Vivo*

Mice were euthanized and immediately intubated. Bronchoalveolar lavage fluid (BALF) was collected as previously reported (22). Briefly, the lungs were immediately lavaged using a tracheal cannula with 1 ml of phosphate-buffered saline (PBS); BALF was collected after five bouts of pumping. Bronchoalveolar lavage was repeated three times for each mouse. The BALF was centrifuged at 4°C for 10 min and the supernatant was collected for further analysis. Evans blue dye (EB, Cat. No: E2129, Sigma, Germany) in PBS was injected *via* the tail vein at a dose of 30 mg/kg, then the lungs were lavaged with PBS after 1 hour. BALF was collected and quantitated in a micro‐plate reader by measuring absorbance at 620 nm as previously described (23). EB leakage was calculated according to a standard curve.

### Bacterial Translocation and Transwell-EB Dye Monolayer Permeability Assay

Transwell inserts (pore size 3.0 μm, #3472; Costar, Temecula, CA, USA) were used to measure bacterial translocation and monolayer permeability as previously described (24). Briefly, epithelial cells were seeded in Transwell inserts and incubated for 5–7 days; TEER was measured to monitor the monolayer integrity. Bacterial cells were washed three times in PBS and resuspended in FBS-free DMEM at an MOI of 50 and were added to the apical side of the Transwell inserts, and after incubated for 3 h at 37°C in 5%CO_2_, culture medium in the lower chamber was collected, then diluted and spread on MH-Agar plates. Culture medium in the upper chamber was replaced with EB-conjugated albumin (EBA, final concentration: 0.67 mg/mL). The mixture in the lower chamber was collected after incubation for 1 h and quantified in a microplate reader *via* measurement of OD_620_.

### Enzyme-Linked Immunosorbent Assay (ELISA)

The BALF and peripheral blood serum were collected from mice and stored at −80°C. The levels of TNF-α and IL-6 were determined using ELISA kits(Cat. No. 70-EK206/3, 70-EK282/4; MultiSciences Biotech, China), in accordance with the manufacturer’s recommendations.

### Wound Healing Assay

The wound healing assay protocol was performed as previously described (24). Briefly, A549 and HBE cells were seeded in tissue culture plates and grown to confluence. Cell monolayers were scraped using a 200 μL pipette tip at 6 h after infection with *A. baumannii*, then washed with PBS to clear cell debris. Complete medium was removed and replaced with DMEM supplemented with 1% serum and 20 μg/mL gentamycin. The cells were then subjected to other treatments or directly incubated for 24 h at 37°C in an atmosphere of 5% CO_2_. Images of the wound were captured under a microscope at 0 and 24 h in the same position. Cell migration ability was determined by the rates of scratch wound confluence using Adobe Photoshop 2018 (Adobe Systems Inc., San Jose, CA, USA).

### Transepithelial Electric Resistance Measurements

Epithelial barrier integrity was determined by measurement of electrical resistance (Millicell-ERS, MERS00002; Merck Millipore, Darmstadt, Germany). Cells were seeded in Transwell inserts (pore size 0.4 μm, #3470; Costar) as previously described; the electric resistance of A549 cell monolayers was measured at 2-h intervals. Transepithelial electric resistance (TEER) was calculated in accordance with the manufacturer’s recommendations; a higher TEER was indicative of greater barrier integrity.

### Immunofluorescence Staining and Confocal Microscopy

The cells were treated as previously described (24). Briefly, cells or tissue slices were incubated overnight at 4°C with primary antibodies (**Supplementary Table 3**). After cells had been washed three times, they were incubated with secondary antibodies for 1 h. Cells or tissue slices were again washed with PBS and incubated with 4′,6-diamidino-2-phenylindole (DAPI) for 10 min. Finally, the cells were washed three times in PBS and observed under a confocal microscope (A1-Ti; Nikon, Tokyo, Japan).

### RNA Preparation and Quantitative Reverse Transcription Polymerase Chain Reaction (qRT-PCR)

Total RNA was extracted and RT-PCR was performed in accordance with the manufacturer’s instructions (Cat. No. RR037A; TaKaRa, Tokyo, Japan). The degree of induction was calculated using the cycle threshold (Ct) method. qPCR was performed with an initial denaturation step at 95°C for 30 s, followed by 40 cycles of 95°C for 5 s and 60°C for 30 s. TB Green Premix Ex Taq II (Cat. No. RR820A; TaKaRa) was used for signal detection, followed by real-time PCR analysis (Roche LightCycler480; Roche Molecular Systems, Basel, Switzerland) to examine gene expression patterns in live tissue and cells. Primer sequences are listed in **Supplementary Table 4**.

### Western Blotting

Total protein was extracted as previously described (24). Briefly, mouse tissue and A549 cells was homogenised by grinding and ultrasonication, and the protein concentrations were then measured. Protein samples were separated by sodium dodecyl sulphate-polyacrylamide gel electrophoresis (SDS-PAGE) and transferred onto polyvinylidene difluoride membranes. After blots had been blocked, they were incubated overnight with primary antibodies. All membranes were stained with a secondary antibody, developed using an enhanced chemiluminescence kit (Cat. No. WBKLS0050; Merck Millipore), and photographed using a chemiluminescence imaging system (ChemiDoc MP, Bio-Rad, Richmond, CA, USA).

### Co-Immunoprecipitation and Co-Localisation Experiments

Co-immunoprecipitation (Co-IP) and co-localisation experiments were performed as previously described (25). In the Co-IP experiments, Flag-tagged *ompA* (pCMV-Flag-*ompA*) was co-transfected into HEK293 cells along with either pEGFP-N1 vector or GFP-tagged *tlr2* (pEGFP-*tlr2*). At 48 h after transfection, the cells were lysed in 250 μL of ice-cold lysis buffer. The cell lysates were incubated with anti-Flag antibody overnight at 4°C with rotation. After incubation, 20 μL of protein A magnetic beads (Cat. No. 161-4013; Bio-Rad) were added to the cell lysates and incubated at 4°C for 1 h with rotation. The beads were then washed in wash buffer and boiled in loading buffer. The eluted proteins were separated by SDS-PAGE, transferred onto polyvinylidene difluoride membranes, and detected with anti-GFP antibody.

A549 cell lysates were incubated with rabbit IgG or primary antibodies overnight at 4°C with rotation. After incubation, protein A magnetic beads were added and incubated for an additional 1 h at 4°C with rotation. After incubation, the beads were washed and the eluted proteins were treated as previously described (24), they were then detected by Western blotting with primary antibodies. The associations between β-catenin and α-catenin or primary antibodies were quantified. The binding ability of β-catenin/IQGAP1, β-catenin/α-catenin and E-Cadherin/β-catenin were expressed as the ratio of β-catenin (IP) to IQGAP1 (IP), β-catenin (IP) to α-catenin (IP) and E-Cadherin (IP) to β-catenin (IP).

For co-localisation experiments, HEK293 cells were seeded on cover glasses and co-transfected with pCMV-Flag-*ompA* and either pEGFP-N1 vector or pEGFP-TLR2. At 48 h after transfection, the cells were fixed with 4% paraformaldehyde for 15 min, then permeabilised in 0.1% Triton X-100 at room temperature. Next, immunofluorescence staining was performed using anti-Flag rabbit antibody and anti-GFP mouse antibody; reactions were visualised by confocal microscopy.

### Statistical Analysis

The data are expressed as means ± standard deviations. Comparisons between two groups were performed using two-tailed unpaired Student’s *t*-tests. One-way analysis of variance was used for multiple-group comparisons. Two-way repeated measures analysis of variance was used to compare changes in a single variable over time in each group. Statistical analyses were performed using SPSS 23.0 (SPSS Inc., Chicago, IL, USA) and GraphPad Prism 7.0 (GraphPad Software, San Diego, CA, USA). In all analyses, *P* < 0.05 was considered to indicate statistical significance.

## Results

### OmpA Contributes to *A. baumannii* Translocation Across Epithelial Cell Monolayers and Intrapulmonary Bacterial Load

**Figure 1:**
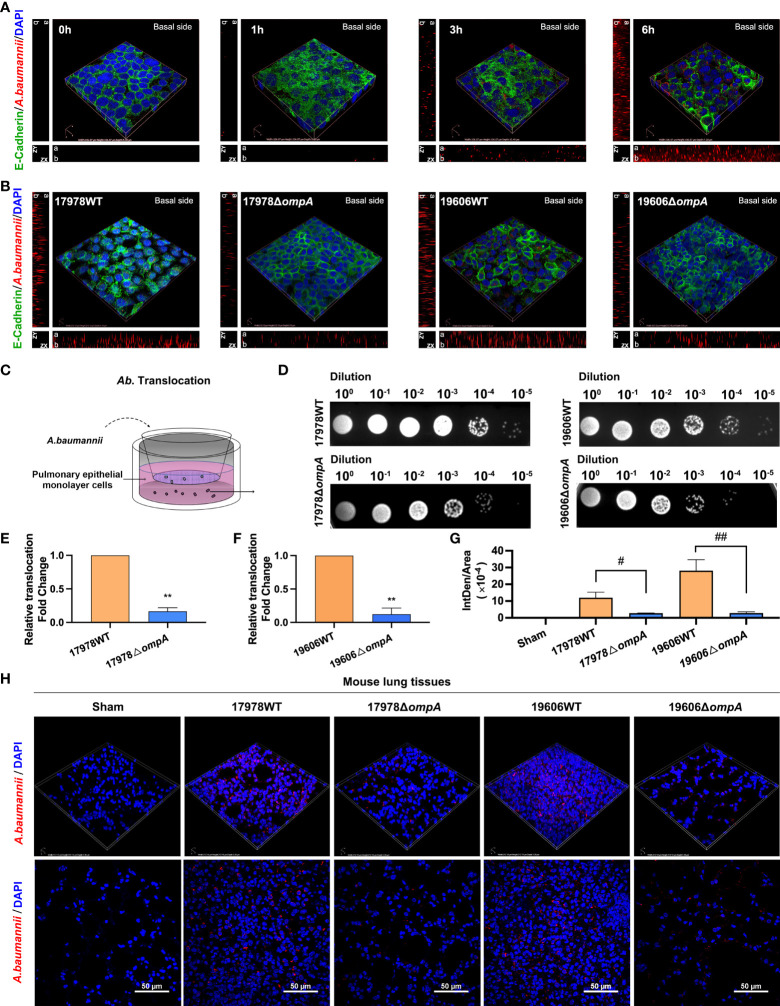
Different *Acinetobacter baumannii* strains’ translocation across monolayer epithelial cells and intrapulmonary bacterial load. **(A)** Translocation of *A. baumannii* ATCC17978 at multiple time points. Red fluorescence represents *A. baumannii* on the basal side of the A549 cell monolayer. ZX and ZY represents z-stack images, “a” represents apical side and “b” represents basal side. **(B)** Translocation of WT *A baumannii* (ATCC17978 and ATCC19606) and corresponding Δ*ompA* deletion strains across the A549 cell barrier at 6 h after infection. **(C)** Schematic graph of the transwell translocation assay. **(D)** Distributions of different *A. baumannii* strains in the lower Transwell chambers at 6 h after infection. **(E, F)** *A. baumannii* WT and their Δ*ompA* deletion strains translocation across A549 cell barrier. **(G, H)** The burden of different *A. baumannii* strains in mouse lung tissue at 48 h after challenge (*n* = 3 per group). **(G)** Mean optical density of different *A. baumannii* in mouse lung tissue. Red fluorescence represents the *A. baumannii*, Green fluorescence represents the E-Cadherin and blue fluorescence represents the nuclei, scale bar, 50μm. **P<0.01 vs. 17978WT/19606WT group, ^##^ *P*<0.01, ^#^ *P*<0.05.

WT and *ompA* deletion *A. baumannii* strains were used to infect A549 cells and challenge C57BL/6J mice. Bacterial translocation abilities were evaluated by immunofluorescence and Transwell translocation assays. As expected, a significant increase in the fluorescence intensity of *A. baumannii* was observed on the basal side of A549 cell monolayer with increasing infection times (**Figure 1A**; **Figure S1A**). Next, A549 cells were infected with the WT and *ompA* deletion *A. baumannii* strains for 6 h at an MOI of 50, in addition, the differences of bacterial burden within 6 h between WT/*ompA* deletion strains in cell infection model were excluded (**Figures S3D–G**). The fluorescence intensity of *A. baumannii* on the basal side of A549 cell monolayer was higher in the WT infection groups compared to the Δ*ompA* infection groups (**Figure 1B**; **Figure S1B**); furthermore, the *ompA*-complemented strains (Δ*ompA ompA* ^+^) showed restoration of translocation in the A549 cell monolayer (**Figure S1C**). In the Transwell translocation assay, the numbers of bacteria crossing the epithelial cell monolayer follows the same trend as those of the immunofluorescence analyses (**Figures 1C–F**). We also investigated the effects of *A. baumannii* in the lung tissue of mice challenged by intratracheal injection of WT and Δ*ompA* strains after 48 h. Compared to the WT strains, immunofluorescence analysis of tissue sections showed decreased bacterial burden in epithelial cells of mice that had been challenged with the Δ*ompA* strains (**Figures 1G, H**); the *ompA*-complemented strains (Δ*ompA ompA* ^+^) also showed increased intrapulmonary burden in mice (**Figure S1C**).

### OmpA^+^ *A. baumannii* Increased Pulmonary Permeability and Promoted Epithelial Barrier Dysfunction

**Figure 2:**
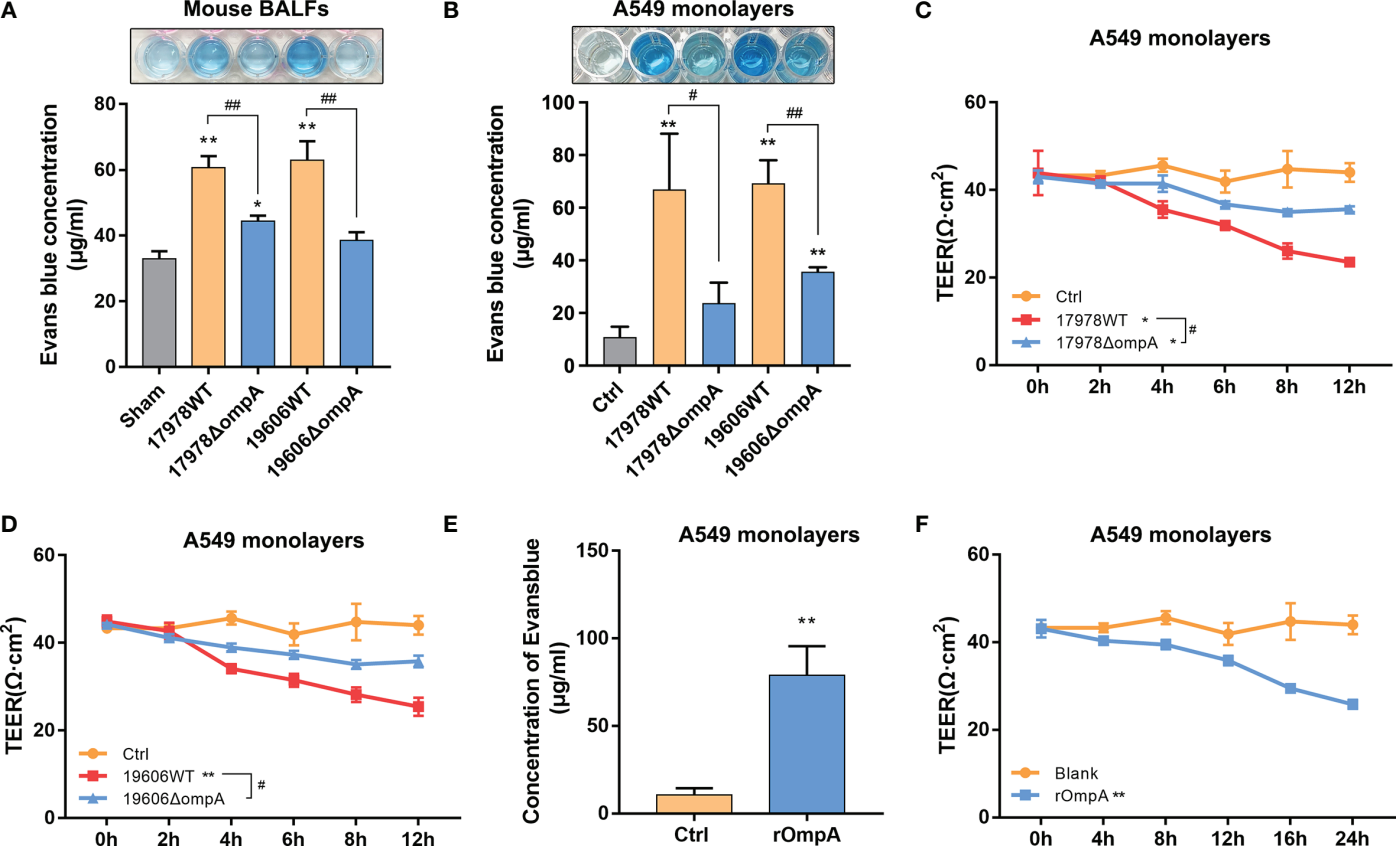
Outer membrane protein A (OmpA)^+^ *A. baumannii* increased pulmonary permeability and promoted epithelial barrier dysfunction. **(A)** Evans blue dye (EB) permeability through the pulmonary epithelium of uninfected (Sham) and different *A. baumannii* strain-infected C57BL/6J mice measured in bronchial alveolar lavage fluid (BALF) (*n* = 3 per group). **(B)** EB-conjugated albumin (EBA, final concentration: 0.67 mg/mL) flux after infection with different *A. baumannii* strains (MOI = 50). **(C, D)** TEER of A549 cell monolayers at multiple time points after treatment with different *A. baumannii* strains. **(E)** Purified OmpA pre-treatment affected the EBA permeability and **(F)** TEER of A549 cells at multiple time points. Data are from three independent experiments; error bars represent standard deviation. **P<0.01,*P<0.05 vs. Control (or Sham, blank) group, ^##^ *P*<0.01, ^#^ *P*<0.05.

Next, we analysed pulmonary permeability using the paracellular marker Evans blue (EB). Briefly, *A. baumannii*-infected mice were sacrificed 1 h after EB injection through the tail vein. Relative to uninfected controls, the EB concentrations were significantly increased in the BALF of mice at 48 h after intratracheal challenge with the WT strains, while the levels in Δ*ompA* strain-challenged mice were significantly decreased (**Figure 2A**). We also evaluated the A549 monolayer permeability of WT and Δ*ompA* strains by EB-conjugated albumin (EBA) leakage and transepithelial electric resistance (TEER) measurements. Leakage of EBA from the upper chamber was significantly increased in the WT strain-infected groups, compared to the control and Δ*ompA* strain-infected groups (**Figure 2B** **)**. As expected, TEER showed a trend opposite to EB leakage **(** **Figures 2C, D**). To define the role of bacterial OmpA, we treated epithelial cells with purified recombinant OmpA (rOmpA, purity has been verified, see **Figure S3H**). Treatment with rOmpA protein significantly increased the EB leakage and progressively reduced the TEER of A549 monolayers with increasing OmpA treatment time (**Figures 2E, F**). Nonspecific effects of purified protein were excluded by using bovine serum albumin as a control (**Figures S3I, J**). In addition, the presence of OmpA did not affect cell viability, as demonstrated by CCK8 assays (**Figure S1D**). Challenge with Δ*ompA*- complemented strains increased EB leakage *in vivo* and *in vitro*; it also reduced the TEER of A549 cell monolayers (**Figure S1E–G**).

### OmpA^+^ *A. baumannii* Upregulated TNF-α and IL-6 Expression Levels in Epithelial Cells and Mouse Lungs

**Figure 3:**
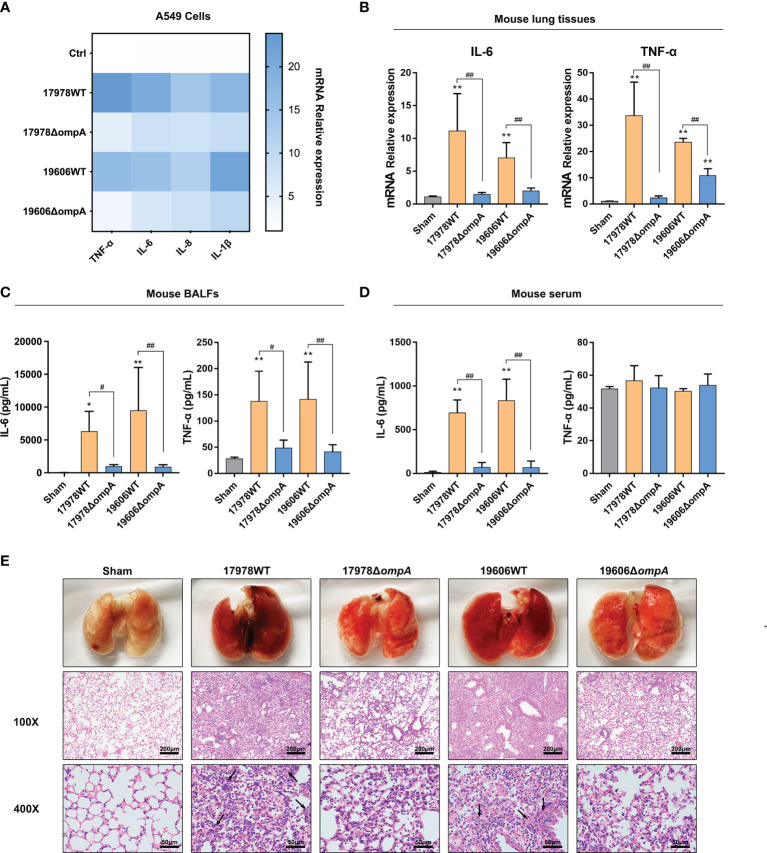
OmpA^+^ *A. baumannii* upregulated TNF-α and IL-6 expression levels in epithelial cells and mouse lungs. **(A)** TNF-α, IL-6, IL-8, and IL-1β mRNA levels in A549 cells at 6 h after infection with different *A baumannii* strains. **(B)** IL-6 and TNF-α mRNA levels in mouse lung tissue challenged with different *A. baumannii* strains at 48 h after infection (*n* = 3 per group). **(C, D)** ELISA of IL-6 and TNF-α in mouse (*n* = 5 per group) BALF **(C)** and serum **(D)** at 48 h after challenge with different *A. baumannii* strains. **(E)** Representative haematoxylin and eosin (HE)-stained images from uninfected mice (Sham) and mice infected with different *A. baumannii* strains. Alveolar cavity shrinking, septum thickening, interstitial diffuse oedema (arrows) are evident (*n* = 3 per group), scale bar, 200μm (100X), 50μm (400X). **P<0.01,*P<0.05 vs. Sham group, ^##^ *P*<0.01, ^#^ *P*<0.05.

We determined the mRNA levels of pro-inflammatory cytokines, including TNF-α, IL-6, IL-8 and IL-1β, which have been shown to affect homeostasis of the pulmonary epithelial barrier and induce epithelial barrier dysfunction. In both A549 cells and *A. baumannii*-infected mouse lung tissue, the levels of TNF-α and IL-6 transcripts (also including IL-8 and IL-1β transcripts in A549 cells) were significantly upregulated in the groups infected with the WT strains, compared to the controls and groups infected with the Δ*ompA* strains (**Figures 3A, B**; **Figure S1N**), Next, ELISA was performed to determine the levels of IL-6 and TNF-α in mouse BALF and peripheral blood serum. The protein expression levels of IL-6 and TNF-α in mouse BALF were indicative of the respective mRNA levels in mouse lung tissue and A549 cells (**Figure 3C**). Only the level of IL-6 in peripheral blood serum differed between groups infected with the WT strains and groups infected with the Δ*ompA* strains (**Figure 3D**); the Δ*ompA*- complemented strains showed restored pro-inflammatory capacity according to findings in serum and BALF samples (**Figures S1H–K**). Histopathological analysis of mouse lung tissue revealed significant diffuse inflammatory cell infiltration, alveolar cavity shrinking, septum thickening, interstitial diffuse oedema, cell arrangement disorder, and tissue integrity damage in mice challenged with the WT strains, compared to mice challenged with the Δ*ompA* strains (**Figure 3E**).

### OmpA Contributes to *A. baumannii*-Induced TLR2/NF-κB Activation for Increased Epithelial Permeability

**Figure 4:**
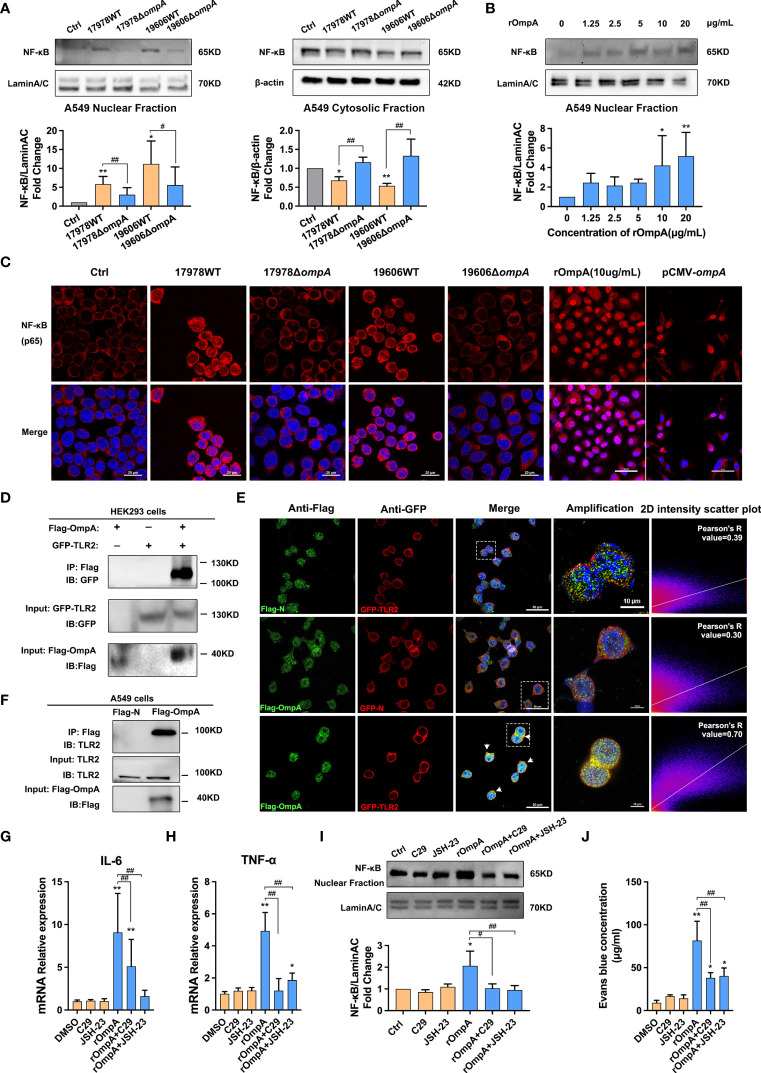
OmpA contributes to *A. baumannii*-induced TLR2/NF-κB activation for increased epithelial permeability. **(A)** Distributions of NF-κB in the cytoplasm and nucleus were evaluated by Western blotting. Relative NF-κB protein levels in the nuclei and cytoplasm are expressed relative to Lamin A/C and β-actin, respectively. **(B)** Distributions of NF-κB in the nuclei of A549 cells treated with different concentrations of purified OmpA (rOmpA). **(C)** The effects of different *A. baumannii* strains, rOmpA, and pCMV-*ompA* plasmid transfection on localisation of NF-κB were evaluated by immunofluorescence labeling, Red represents NF-κB and blue indicates nuclei. Scale bar: 20 μm. **(D)** Binding of OmpA-Flag and TLR2-GFP in HEK293 cells. **(E)** Co-localisation of OmpA-Flag and TLR2-GFP proteins was detected by double immunofluorescence staining in HEK293 cells, green represents Flag/OmpA-Flag, red represents GFP/TLR2-GFP, yellow represents OmpA-Flag/TLR2-GFP co-localization and blue indicates nuclei, scale bar: 50μm. Amplified images are shown below, scale bar: 10μm. **(F)** OmpA-Flag and TLR2 in A549 cells were detected by co-immunoprecipitation assay. **(G–J)** The effects of the TLR2 inhibitor, C29, and NF-κB inhibitor, JSH-23, on mRNA levels of IL-6 **(G)** and TNF-α **(H)**, distribution of NF-κB in the nucleus **(I)**, and EBA leakage **(J)** in A549 cells treated with rOmpA. **P<0.01,*P<0.05 vs. Control (or blank, DMSO) group, ^##^ *P*<0.01, ^#^ *P*<0.05.

NF-κB signaling is a crucial regulator of pro-inflammatory cytokines, including IL-6 and TNF-α. Therefore, we examined whether OmpA contributed to NF-κB activation. Western blotting and immunofluorescence analyses were performed to assess the distribution of NF-κB in the cytoplasm and nucleus. Western blotting showed significantly lower levels of nuclear NF-κB in control A549 cells and cells infected with the Δ*ompA* strains, compared to levels in cells infected with the WT strains; the levels of cytosolic NF-κB showed the opposite trend (**Figure 4A**). Treatment with purified rOmpA protein also increased the NF-κB level in the A549 cell nucleus in a dose-dependent manner (**Figure 4B**). Immunofluorescence analysis of the nuclear localisation of NF-κB revealed results that were similar to the findings of Western blotting. Transfection with pCMV-*ompA* also increased NF-κB activation (**Figure 4C**), indicating that OmpA contributes to *A. baumannii*-induced NF-κB activation in A549 cells. Because TLR2 is a possible target for OmpA, we analysed the possible relationship between OmpA and TLR2. We constructed eukaryotic expression vectors of OmpA and TLR2 tagged with Flag and GFP, respectively (pCMV-Flag-*ompA* and pEGFP-*tlr2*, respectively); these were co-transfected into HEK293 cells. Co-IP revealed interactions between OmpA-Flag and TLR2-GFP (**Figure 4D**). Double immunofluorescence staining revealed co-localisation of OmpA-Flag and TLR2-GFP proteins in HEK293 cells (**Figure 4E**). pCMV-Flag-*ompA* was also transfected into A549 cells; co-IP also confirmed the interaction in these cells (**Figure 4F**). Next, we examined the effects of C29 (a specific inhibitor of TLR2) and JSH-23 (an inhibitor of NF-κB) on OmpA-induced inflammation activation and epithelial monolayer permeability. Both 50 μM C29 treatment and 20 μM JSH-23 treatment significantly inhibited rOmpA protein-induced NF-κB activation and downregulated the expression levels of IL-6 and TNF-α (**Figures 4G–I**), while reducing EBA leakage and restoring A549 cell barrier function (**Figure 4J**), which may be caused by the inhibition of adherent junction cleavage (**Figure S3B** **)**. In addition, there were no significant differences in NF-κB activation, IL-6 or TNF-α expression, or EB leakage between groups treated with either C29 or JSH-23 and the controls (**Figures 4G–J**), CCK8 assays showed that neither C29 nor JSH-23 affected cell viability (**Figures S1L, M**).

### OmpA Induces Cytoskeleton Rearrangement and AJ Internalisation for Increased Epithelial Permeability and *A. baumannii* Translocation

**Figure 5:**
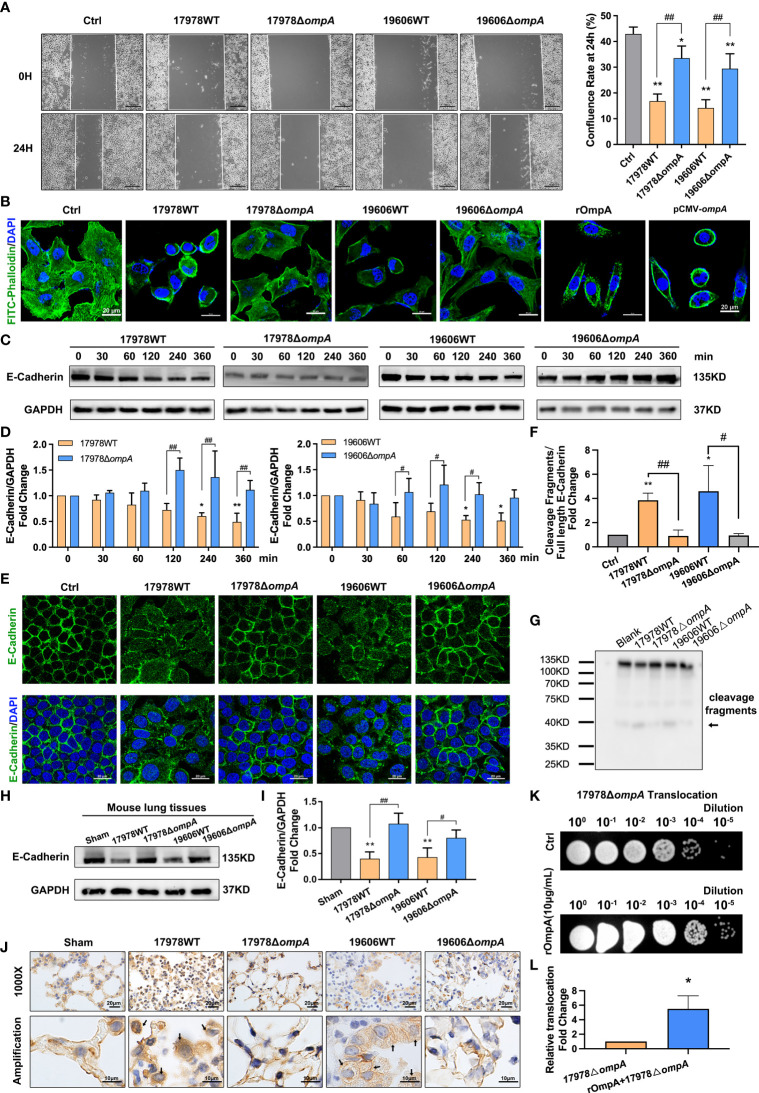
OmpA induces cytoskeleton rearrangement and AJ internalisation for increased epithelial permeability and *A. baumannii* translocation. **(A)** Quantification of confluence rate of A549 cells at 24 hours after infection which represent the migration ability [% wound confluence = (a − b) × 100%/a; a = Initial scratch wound area at 0 h, b = Scratch wound area at 24 h], scale bar, 200μm. **(B)** FITC-phalloidin staining showed the cytoskeletal changes in A549 cells infected with different *A. baumannii* strains, treated with purified OmpA, or transfected with pCMV-*ompA* expression plasmid. Green represents phalloidin, and blue indicates nuclei. Scale bar: 20μm. **(C, D)** Expression of the adherens junction protein, E-cadherin, was evaluated by Western blotting. E-cadherin protein levels are expressed relative to GAPDH. **(E)** Localisation of E-cadherin was evaluated by immunofluorescence labeling. Green represents E‐cadherin and blue indicates nuclei. Scale bar: 20μm. **(F, G)** E-cadherin cleavage fragments (arrows) detected by the western blot, cleavage fragments levels are expressed relative to full length protein (135KD). **(H–J)** The expression level **(H, I)** and cellular localisation **(J)** of E-cadherin in the lung tissue of mice challenged by intratracheal injection of different *A baumannii* strains. (*n* = 3 per group), scale bar, 20μm (1000X), 10μm (Amplification). **(K, L)** Translocation of Δ*ompA* strains across the A549 cell monolayer pre-treated with rOmpA protein. **P<0.01,*P<0.05 vs. Control (or Sham, blank, 17978△*ompA*) group, ^##^ *P*<0.01, ^#^ *P*<0.05.

To analyse changes in cytoskeleton function, cell migration ability was examined using wound healing assays. The results indicated a time-dependent decrease in migration ability in A549 cells that had been infected with WT *A. baumannii*; significant inhibition was observed after 6 h (**Figures S2A, B**). Next, we examined the cell migration abilities of A549 cells infected with WT and Δ*ompA* strains. Wound healing assays showed that the confluence rate at 24 h, which represents migration ability, was significantly reduced in the groups infected with the WT strains, compared to the uninfected controls and the groups infected with the Δ*ompA* strains (**Figure 5A**). Immunofluorescence staining with FITC-phalloidin showed regularly arranged actin fibres beneath the cell membrane in the uninfected group. Obvious skeletal remodeling (disorderly arrangement, different thicknesses, unevenly distributed microfilaments, and missing cytoskeleton) was observed in the groups infected with the WT strains. A549 cells infected with the Δ*ompA* strains showed less marked changes in cytoskeletal structure than did A549 cells infected with the WT strains (**Figure 5B**). In addition, A549 cells treated with rOmpA protein or transfected with pCMV-*ompA* plasmid showed similar changes in cytoskeletal structure (**Figure 5B**). The expression level of E-cadherin, a classic cell AJ protein, was downregulated over time in the groups infected with the WT strains, compared to the controls and groups infected with the Δ*ompA* strains (**Figures 5C, D**). Laser scanning confocal microscopy showed obvious E-cadherin internalisation from the A549 and HBE cell membrane into the cytoplasm in groups infected with the WT strains (**Figure 5E**, **Figure S2C**). E-cadherin cleavage fragments were also detected by the western blot and follows the same trend as those of the immunofluorescence analyses (**Figures 5F, G**). The expression level and cellular localisation of E-cadherin in the lung tissue of mice challenged by intratracheal injection of WT and Δ*ompA A. baumannii* strains were examined after 48 h of infection. As expected, relative to the uninfected control and Δ*ompA* strain-infected groups, E-cadherin showed significant downregulation and internalisation in the groups infected with the WT strains (**Figures 5H–J**, **Figure S3A**). A549 cells transfected with pCMV-*ompA* also showed trends similar to the findings in WT strain-infected groups (**Figure S2D**). Furthermore, the translocation of the Δ*ompA* strains across the epithelial cell monolayer was significantly increased in A549 cells that had been pre-treated with rOmpA protein (**Figures 5K, L**).

### OmpA-Dependent Inflammatory Regulation and Cytoskeleton/Cell Junction Dysfunction Require IQGAP1 in Epithelial Cells

**Figure 6:**
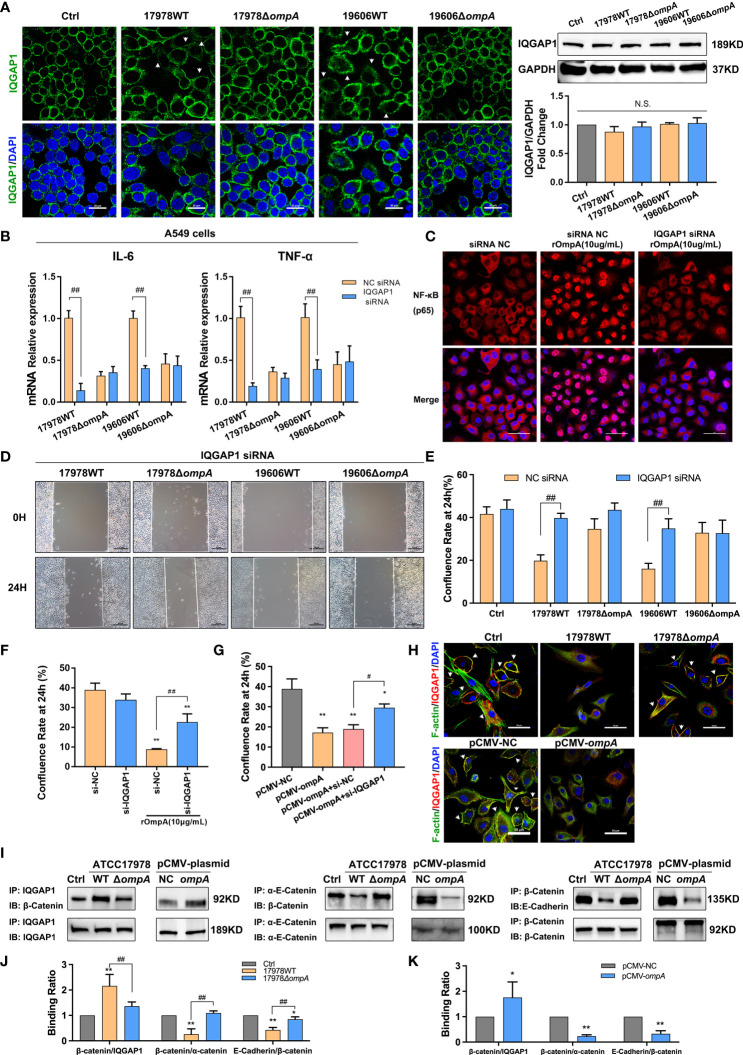
OmpA-dependent inflammatory regulation and cytoskeleton/cell junction dysfunction require IQGAP1 in epithelial cells. **(A)** Redistribution of IQGAP1 was evaluated by immunofluorescence labeling (arrows). Green represents IQGAP1 and blue indicates nuclei. Scale bar: 20 μm. Expression of IQGAP1 was evaluated by Western blotting. The IQGAP1 protein level is shown relative to GAPDH. **(B, C)** The effects of IQGAP1 knockdown on IL-6 and TNF-α expression level **(B)** and NF-κB activation **(C)** in A549 cells infected with different *A. baumannii* strains. Red represents NF-κB and blue indicates nuclei. Scale bar: 50 μm. **(D–G)** The effects of IQGAP1 knockdown on migration ability of A549 cells infected with different *A. baumannii* strains **(D, E)**, treated with rOmpA **(F)**, or transfected with pCMV-*ompA* expression plasmid **(G)**. **(H)** The co-localisation of IQGAP1 and F-actin was detected by immunofluorescence; the interaction of IQGAP1 with E-cadherin–catenin complex in A549 cells was detected by co-immunoprecipitation assay, green represents F-actin, red represents IQGAP1, yellow represents IQGAP1/F-actin co-localization (arrows) and blue indicates nuclei, scale bar, 50μm. **(I–K)** The binding ability of β-catenin/IQGAP1, β-catenin/α-catenin and E-Cadherin/β-catenin in A549 cells infected with different *A baumannii* strains or transfected with pCMV-*ompA* expression plasmid were expressed as the ratio of β-catenin (IP) to IQGAP1 (IP), β-catenin (IP) to α-catenin (IP) and E-Cadherin (IP) to β-catenin (IP). **P<0.01,*P<0.05 vs. Control (or NC siRNA, pCMV-NC) group, ^##^ *P*<0.01, ^#^ *P*<0.05; N.S., no significance.

Immunofluorescence staining showed that the scaffold protein IQGAP1 was significantly depleted from the cell membrane of WT strain-infected A549 and HBE cells, compared with the Δ*ompA* strain-infected groups and controls, without changes in protein expression level (**Figure 6A**; **Figure S2E**). pCMV-*ompA* plasmid transfection also showed trends similar to the findings in WT strain-infected groups (**Figures S2F, G**). RNA interference assays were performed to assess host factors involved in *A. baumannii* infection; particularly eukaryotic factors related to OmpA-dependent signaling. Here, we studied the roles of IQGAP1 in cytoskeletal dynamics. qPCR and Western blotting analysis showed that the IQGAP1 expression level was significantly decreased after treatment with IQGAP1-targeting small interfering RNA (**Figures S2H, I**). qPCR showed that IQGAP1 depletion prevented the upregulation of IL-6 and TNF-α induced by infection with the WT strains but did not affect IL-6 and TNF-α expression in the Δ*ompA* strain-infected groups (**Figure 6B**). Immunofluorescence analysis showed that IQGAP1 knockdown reduced the nuclear localisation of NF-κB activated by rOmpA (**Figure 6C**). IQGAP1 knockdown reduced the inhibition of cell migration mediated by OmpA, but did not significantly affect the confluence rate of untreated cells in wound healing assays (**Figures 6D, E**, **S3B**). IQGAP1 knockdown also ameliorated the inhibition of cell migration induced by rOmpA pre-treatment or pCMV-*ompA* transfection (**Figures 6F, G**). These results suggested that IQGAP1 may mediate OmpA signal transduction in host cells. Next, to investigate whether OmpA modulates the interaction of IQGAP1 with related intracellular proteins, A549 cells infected with the WT or Δ*ompA A. baumannii* strains, or transfected with pCMV-*ompA* plasmid, were examined by co-IP and co-localisation assays. The assays examined interactions of F-actin (an important cell migration-related protein) and α-catenin/β-catenin (related to E-cadherin stability in the membrane) with IQGAP1. Both infection with WT strains and transfection with pCMV-*ompA* plasmid inhibited the interaction between IQGAP1 and F-actin (**Figure 6H**); they also promoted the interaction between IQGAP1 and β-catenin, thus inducing the dissociation of α-catenin from the E-cadherin–catenin complex. These changes eventually led to the inhibition of cell migration and internalisation of E-cadherin (**Figures 6I–K**).

## Discussion

*A. baumannii* causes nosocomial infections (e.g., pneumonia and catheter-related bloodstream infections in intensive care unit patients), as well as soft tissue and urinary tract infections. Antibiotic resistance rates of *A. baumannii* strains have increased in recent years; effective treatment is thus a major clinical challenge (5). There is an urgent need to elucidate the pathophysiological mechanism of *A. baumannii* infection, but the mechanism by which *A. baumannii* crosses the pulmonary epithelial barrier during the infection phase has not yet been fully elucidated. Previous studies showed that the overexpression of OmpA in *A. baumannii* is a risk factor for nosocomial pneumonia, bacteraemia, and increased mortality rate (6). Infection experiments *in vivo* showed that *A. baumannii* strains expressing high levels of OmpA were more likely to cause bacteraemia in mice; such infections were positively correlated with the level of IL-6 in mouse blood (26). Considering the toxic effect of OmpA on host cells, we speculated that it may be involved in the dissemination and deterioration of *A. baumannii* pulmonary infection. In this study, we used an *in vivo* mouse model and *in vitro* epithelial cell monolayer/alveolar epithelial barrier model to establish that OmpA contributes to the dissemination of *A. baumannii* in mouse lungs and translocation across the alveolar epithelial cell monolayer. Epithelial barrier function is critically dependent on barrier integrity (27). Many infections (viral, bacterial, and fungal) also compromise the barrier integrity of the airway epithelium, increase epithelial permeability, and lead to a loss of barrier function, which are important changes that allow pathogen invasion (28). Using both model systems, we showed that OmpA contributes to high pulmonary epithelial barrier permeability of *A. baumannii* both *in vitro* and *in vivo*. Therefore, we focused on exploring the mechanism of *A. baumannii* OmpA-mediated barrier dysfunction.

Inflammatory responses have important roles in the regulation of epithelial barrier function; specifically, inflammatory mediators (e.g., TNF and ILs) induce inflammatory injury and repair of the epithelial barrier (29, 30), which may be involved in cytoskeleton dynamics and the regulation of cell–cell junctions (31). Transcriptome profiling revealed that genes related to TNF, cytokine-cytokine receptor interaction, and TLR were significantly upregulated in *A. baumannii* acute lethal pneumonia (32). Therefore, we examined the expression levels of pro-inflammatory cytokines; we found that *A. baumannii* OmpA significantly upregulated the expression levels of IL-6 and TNF-α in both *A. baumannii*-infected mouse lung tissue and in cultured A549 cells. In addition, histopathological analysis showed that OmpA^+^ WT *A. baumannii* strains caused more significant inflammatory pathological changes in mouse lung tissue than did the OmpA^−^ deletion mutant Δ*ompA* strain, indicating a strong pro-inflammatory role of OmpA. NF-κB is a crucial signaling molecule involved in the regulation of pro-inflammatory cytokines, including TNF-α and IL-6 (33). We examined whether *A. baumannii*-OmpA contributes to NF-κB (p65) activation; we observed significantly lower levels of nuclear p65 in cells infected with the Δ*ompA* strains than in cells infected with the WT strains. Purified rOmpA protein and eukaryotic expression vector (pCMV-*ompA*) also upregulated the level of p65 in the nucleus.

TLR-mediated NF-κB activation is an ancient defence system that protects the host organism from microbial invasion and subsequent proliferation (34). Early research confirmed that OmpA from *Klebsiella pneumoniae* activates macrophages and dendritic cells in a TLR2-dependent manner (35). There is increasing evidence to support a role for OmpA in the activation of TLR2, as well as a potential interaction between OmpA and TLR2 (12, 36). In this study, we showed that OmpA was a potential TLR2 binding candidate in *A. baumannii*. Because TLR2 is a pattern recognition receptor, its activation triggers downstream signal transduction, induces epithelial inflammation through the classical NF-κB pathway (37), and contributes to the regulation of cell function (e.g., cell activation and apoptosis (38), epithelial barrier integrity disruption (39) and cell invasion (40)). We used C29, a specific inhibitor of TLR2, to inhibit TLR2 activation; we found that C29 inhibited the OmpA-mediated upregulation of inflammatory cytokines and nuclear accumulation of NF-κB, while restoring barrier function. JSH-23-mediated inhibition of NF-κB showed a similar effect. Our data suggest that in the infection phase, OmpA activates NF-κB-mediated inflammation through its epithelial receptor, TLR2, thereby contributing to high barrier permeability and bacterial translocation.

In addition to its interactions with receptors on the cell surface, OmpA can enter the cell through outer membrane vesicles (41). Previous studies have shown that OmpA from *Enterobacter sakazakii* is colocalised with microfilaments and microtubules during microbial invasion (42, 43). Our data confirmed that OmpA can injure the cytoskeleton and inhibit the migration of epithelial cells. OmpA also promotes the internalisation and degradation of E-cadherin, a component of AJs; it reduces cell adhesion. In addition to affecting AJs, outer membrane vesicles (OMVs)/outer membrane protein (OMPs) also directly affects other cellular junctions such as tight junctions (TJs), OMVs derived from *Porphyromonas gingivalis* induced cell death with disruption of TJs (include occludins and claudins) in human lung epithelial cells (44). Our previous studies showed that the endothelial cell scaffold protein, IQGAP1, dynamically regulates the actin cytoskeleton during cell migration and maintains cell–cell connections (24); other studies have also confirmed that IQGAP1 is involved in the pathogenesis of multiple pathogens (16, 45). In this study, we observed the IQGAP1-dependent regulation of OmpA. IQGAP1 knockdown significantly inhibited OmpA-mediated inflammation and impaired cell migration; it blocked *A. baumannii* invasion. Earlier studies reported that the endocytosis of trans-interacting E-cadherin is mediated by the IQGAP1–α-catenin/β-catenin complex. Briefly, IQGAP1 binds to β-catenin and causes dissociation of α-catenin from the E-cadherin–catenin complex, leading to E-cadherin instability and weak adhesion (46). As observed in the present study, *A. baumannii* OmpA upregulated the binding of IQGAP1 and β-catenin, dissociated α-catenin from the β-catenin complex, and reduced E-cadherin-mediated cell–cell adhesion. *A. baumannii* OmpA also decreased the interaction between IQGAP1 and F-actin, which has been shown to cause cytoskeletal remodeling (47).

**Figure 7:**
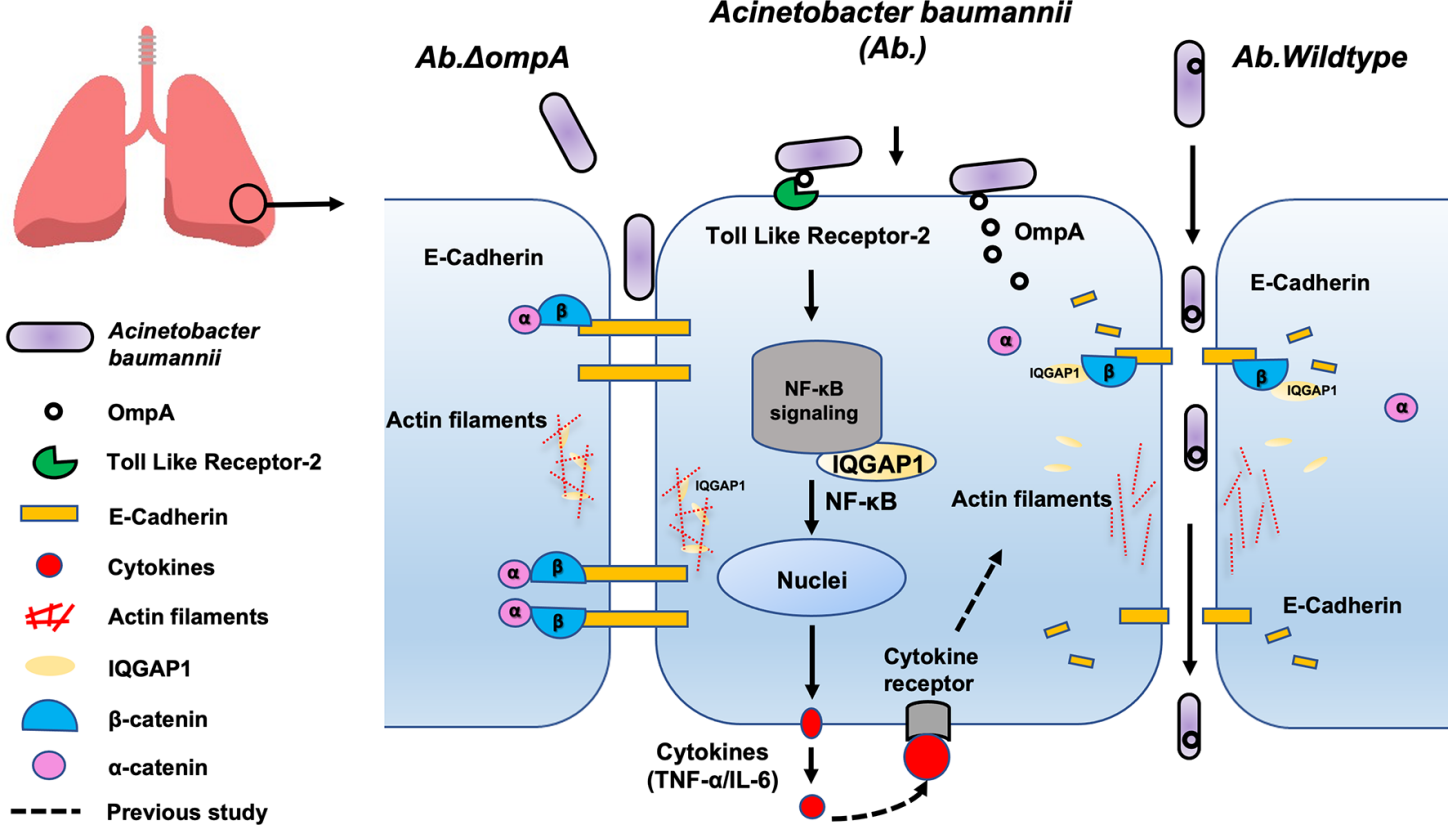
Proposed mechanism involved in pulmonary epithelial barrier dysfunction and bacterial translocation mediated by OmpA in *A. baumannii*. The binding of OmpA to TLR2 activates IQGAP1/NF-κB signaling, which facilitates epithelial cytokine production (TNF-α and IL-6). Moreover, *A. baumannii* OmpA upregulated the binding of IQGAP1 and β-catenin, dissociated α-catenin from the β-catenin complex, and reduced E-cadherin-mediated cell–cell adhesion, it also decreased the interaction between IQGAP1 and F-actin, which led to cytoskeleton dynamic remodeling-associated inhibition of cell migration, these changes led to increased pulmonary permeability and the promotion of epithelial barrier dysfunction and *A. baumannii* translocation.

In conclusion, using an *in vivo* mouse model and an *in vitro* human epithelial cell model, we showed that the binding of OmpA to TLR2 activates IQGAP1/NF-κB signaling, which facilitates epithelial cytokine production (TNF-α and IL-6). Moreover, IQGAP1 mediated OmpA-induced opening of the pulmonary epithelial barrier through the cytoskeleton dynamic remodeling-associated inhibition of cell migration and the cellular redistribution of the major AJ protein, E-cadherin; these changes led to increased pulmonary permeability and the promotion of epithelial barrier dysfunction and *A. baumannii* translocation (**Figure 7**). Our results suggest that OmpA may serve as a therapeutic target for *A. baumannii* infection.

## Data Availability Statement

The original contributions presented in the study are included in the article/**Supplementary Material**. Further inquiries can be directed to the corresponding authors.

## Ethics Statement

The animal study was reviewed and approved by Animal Care and Use Committee at the Sir Run Run Shaw Hospital, College of Medicine, Zhejiang University.

## Author Contributions

WZ and YYu wrote the main manuscript text, XH and SL designed this study, HZ, YJ, JW, and JH participated in part of the experiments, YY and XL analyzed the data. All authors contributed to the article and approved the submitted version.

## Funding

The work was supported by National Natural Science Foundation of China (81871614, 81971897) and National Science Foundation of China International Cooperation and Exchange Programme (81861138054, 32011530116).

## Conflict of Interest

The authors declare that the research was conducted in the absence of any commercial or financial relationships that could be construed as a potential conflict of interest.

## Publisher’s Note

All claims expressed in this article are solely those of the authors and do not necessarily represent those of their affiliated organizations, or those of the publisher, the editors and the reviewers. Any product that may be evaluated in this article, or claim that may be made by its manufacturer, is not guaranteed or endorsed by the publisher.
