# Supplementary material for: Acinetobacter baumannii Outer Membrane Protein A Induces Pulmonary Epithelial Barrier Dysfunction and Bacterial Translocation Through The TLR2/IQGAP1 Axis
Source: Front Immunol. 2022 Jun 30;13:927955. doi: 10.3389/fimmu.2022.927955 (PMC9280087; doi:10.3389/fimmu.2022.927955)
Supplement: Supplementary file 1 [file DataSheet_1.docx]

***Acinetobacter baumannii*** **outer membrane protein A induces** **pulmonary** **epithelial barrier dysfunction and** **bacterial translocation through the TLR2/IQGAP1 axis**

Wang Zhang ^a, b, c, 1^, Hua Zhou ^d, 1^, Yan Jiang ^a, b, c, 1^, Jintao He ^a, b, c^, Yue Yao ^a, b, c^, Jianfeng Wang ^e^, Xiaochen Liu ^a, b, c^, Sebastian Leptihn^a, f, g^, Xiaoting Hua ^a, b, c, *^, Yun song Yu ^a, b, c, *^

^a^ Department of Infectious Diseases, Sir Run Run Shaw Hospital, Zhejiang University School of Medicine, Hangzhou, Zhejiang, P.R. China.

^b^ Regional Medical Center for National Institute of Respiratory Diseases, Sir Run Run Shaw Hospital, Zhejiang University School of Medicine, Hangzhou, China

^c^ Key Laboratory of Microbial Technology and Bioinformatics of Zhejiang Province, Hangzhou, P.R. China.

^d^ Department of Respiratory and Critical Care Medicine, The First Affiliated Hospital, Zhejiang University School of Medicine, Hangzhou, Zhejiang, P.R. China.

^e^ Department of Respiratory and Critical Care Medicine, Zhejiang Provincial Hospital of Chinese Medicine, Hangzhou, Zhejiang, P.R. China.

^f^ Zhejiang University-University of Edinburgh Institute, Zhejiang University, Haining, P.R. China.

^g^ University of Edinburgh Medical School, Biomedical Sciences, College of Medicine & Veterinary Medicine, The University of Edinburgh, Edinburgh, UK

^1^These authors contributed equally to this work.

*Corresponding Author

Yunsong Yu

Department of Infectious Diseases,

Sir Run Run Shaw Hospital, Zhejiang University School of Medicine

3 East Qingchun Road

Hangzhou, Zhejiang, 310016, P.R. China.

Email: yvys119@zju.edu.cn

Xaioting Hua

Department of Infectious Diseases,

Sir Run Run Shaw Hospital, Zhejiang University School of Medicine

3 East Qingchun Road

Hangzhou, Zhejiang, 310016, P.R. China.

Email: xiaotinghua@zju.edu.cn

**Supplementary methods**

**Immunohistochemical staining**

All samples were rinsed in ice-cooled Ringer solution. Mouse lung samples were cut and trimmed to squares and pinned on cork pieces. The tissues were fixed in 4% paraformaldehyde and rinsed in PBS, dehydrated in a graded series of ethanol, embedded in paraffin, cut to 5 µm thin sections, mounted on slides, dewaxed in xylene and rehydrated in a decreasing series of ethanol. Rinsing of the rehydrated sections in 0.01M citrate buffer, pH 6.0. Heat induced epitope retrieval (0.01M citrate buffer, pH 6.0, 96°C, 25s), cooling down of the section container 15s at room temperature and 5s in cold tap water. Rinsing of the sections for 2s, surrounding of sections with a hydrophobic barrier using a barrier pen. Rinsing of the sections in TBS (0.05M Tris-HCl pH 7.6 + 0.9% NaCl). Treated with peroxidase and rinsing in TBS. Preincubated in 3% BSA + 5% normal goat serum in TBS. Incubated in primary antibody overnight at 4°C. Rinsing in TBS, and incubated in HRP labelled goat-anti-rabbit-antibody in the dark. Rinsing in TBS and used DAB for HRP detection. Dehydrated by 100% ethanol and Xylene. Finally added mounting medium and coverslip

**Cell viability assay**

A549 cells were grown in 96-well plates at a density of 10000 cells/well. Once the cells had adhered, different A*cinetobacter baumannii*, C29 or JSH-23 was added to each well and treated for 6h. A Cell Counting Kit-8 (CCK-8; Dojindo, Tokyo, Japan) solution (10 μl) and FBS free medium without P/S (100μl) was then added to each well and incubated for 2hours. Absorbance was then determined at 450nm using a micro-plate reader (Spectra Maxi3x; Molecular Device, CA, USA). We repeated the experiments three times for each group and averaged the results.

**Supplementary Tables**

| **TableS1. Strains used in this Study** |  |  |
| --- | --- | --- |
| Strains | Source | Catalog No |
| *A. baumannii* ATCC17978 | ATCC | #17978 |
| *A. baumannii* ATCC19606 | ATCC | #19606 |
| *A. baumannii* ATCC17978ΔompA | This Study | N/A |
| *A. baumannii* ATCC19606ΔompA | This Study | N/A |
| *E. coli* BL21(DE3) Competent Cells | Vazyme | C504-02 |

| **TableS2. Plasmids used in this Study** | |  |  |
| --- | --- | --- | --- |
| Plasmids | Source | Resistance gene | Catalog No |
| pCMV-Flag-N | Clontech | ampicillin | 635688 |
| p-EGFP-N | Clontech | kanamycin | 6086-1 |
| pYMAb2 | This study | hygromycin | N/A |
| pET28a | Novagen | kanamycin | 69258 |
| pCMV-Flag-ompA | This study | ampicillin | N/A |
| pCMV-ompA | This study | ampicillin | N/A |
| p-EGFP-TLR2 | This study | kanamycin | N/A |
| pYMAb2-ompA | This study | hygromycin | N/A |
| pET28a-ompA | This study | kanamycin | N/A |

| **TableS3. Antibodies used in this Study** | | | | |
| --- | --- | --- | --- | --- |
| Antibodies | Source | | Application | Dilution |
| E‐Cadherin Rabbit antibody | #3195 | Cell Signal Technology | IF/IHC | 1:200 |
|  |  |  | WB | 1:1000 |
| NF-κB Mouse antibody | #6956 | Cell Signal Technology | IF | 1:800 |
| NF-κB Rabbit antibody | #8242 | Cell Signal Technology | WB | 1:1000 |
| IQGAP1 Rabbit antibody | #20648 | Cell Signal Technology | IF | 1:100 |
|  |  |  | WB | 1:1000 |
| β-catenin Rabbit antibody | #8480 | Cell Signal Technology | IP | 1：50 |
|  |  |  | WB | 1:1000 |
| α-catenin Rabbit antibody | #3240 | Cell Signal Technology | IP | 1：50 |
|  |  |  | WB | 1:1000 |
| DYKDDDDK Tag (Flag) Rabbit antibody | #14793 | Cell Signal Technology | IF | 1:100 |
|  |  |  | IP | 1:50 |
|  |  |  | WB | 1:1000 |
| GFP Tag Rabbit Antibody | 50430-2-AP | Proteintech | IF | 1:100 |
|  |  |  | WB | 1:1000 |
| GFP Tag Mouse Antibody | 66002-1-Ig | Proteintech | IF | 1:100 |
|  |  |  | WB | 1:2000 |
| Toll like receptor 2 Rabbit antibody | #12276 | Cell Signal Technology | WB | 1:1000 |
| GAPDH Rabbit Antibody | #5174 | Cell Signal Technology | WB | 1:1000 |
| LaminA/C Rabbit Antibody | #2032 | Cell Signal Technology | WB | 1:1000 |
| *A. baumannii* GnaA Rabbit antibody |  | Huabio | IF | 1:100 |
| CoraLite488-conjugated Goat Anti-Rabbit IgG(H+L) | SA00013‐2 | Proteintech | IF | 1:200 |
| CoraLite594-conjugated Affinipure Goat Anti-Rabbit IgG(H+L) | SA00013‐4 | Proteintech | IF | 1:200 |
| CoraLite594-conjugated Affinipure Goat Anti-Mouse IgG(H+L) | SA00013‐3 | Proteintech | IF | 1:200 |
| HRP-conjugated Goat Anti-Rabbit IgG(H+L) | SA00001-2 | Proteintech | WB | 1:2000 |

| **TableS4. Primers used in this Study** | | | |
| --- | --- | --- | --- |
| Gene | Application |  | Sequence |
| ATCC 17978 ompA | Full length | F: | ATGAAATTGAGTCGTATTGCAC |
|  |  | R: | CATAAAAAAGCGACTCTAACGAGTC |
| Toll like receptor 2 | Full length | F: | ATGCCACATACTTTGTGGATGG |
|  |  | R: | CTAGGACTTTATCGCAGCTCTC |
| IL-6 | qPCR | F: | ACTCACCTCTTCAGAACGAATTG |
|  |  | R: | CCATCTTTGGAAGGTTCAGGTTG |
| TNF-α | qPCR | F: | CCTCTCTCTAATCAGCCCTCTG |
|  |  | R: | GAGGACCTGGGAGTAGATGAG |
| IL-1β | qPCR | F: | ATGATGGCTTATTACAGTGGCAA |
|  |  | R: | GTCGGAGATTCGTAGCTGGA |
| IL-8 | qPCR | F: | ACTGAGAGTGATTGAGAGTGGAC |
|  |  | R: | AACCCTCTGCACCCAGTTTTC |
| GAPDH | qPCR | F: | GGAGCGAGATCCCTCCAAAAT |
|  |  | R: | GGCTGTTGTCATACTTCTCATGG |
| E-Cadherin | qPCR | F: | ATTTTTCCCTCGACACCCGAT |
|  |  | R: | TCCCAGGCGTAGACCAAGA |
| IQGAP1 | qPCR | F: | AGAACGTGGCTTATGAGTACCT |
|  |  | R: | CCAGTCGCCTTGTATCTGGT |

**References**

1. Li Y, Zhu Y, Zhou W, Chen Z, Moran RA, Ke H*, et al.* Alcaligenes faecalis metallo-β-lactamase in extensively drug-resistant Pseudomonas aeruginosa isolates. *Clin Microbiol Infect* 2021.

2. Chamberlain N, Korwin-Mihavics BR, Nakada EM, Bruno SR, Heppner DE, Chapman DG*, et al.* Lung epithelial protein disulfide isomerase A3 (PDIA3) plays an important role in influenza infection, inflammation, and airway mechanics. *Redox biology* 2019, **22:** 101129.

3. Moitra J, Sammani S, Garcia JG. Re-evaluation of Evans Blue dye as a marker of albumin clearance in murine models of acute lung injury. *Transl Res* 2007, **150**(4)**:** 253-265.

**Supplementary Figure legends**

**FigureS1.** (**A**) Translocation of *A. baumannii* ATCC17978 at multiple time points. (**B**) Translocation of WT and corresponding Δ*ompA* deletion strains across the A549 cell barrier at 6 h after infection. (**C**) The *ompA*-complemented strains (Δ*ompA ompA*^+^) showed restoration of translocation in the A549 cell monolayer and intrapulmonary burden in mice at 48 h after challenge (*n* = 3 per group). Red fluorescence represents the *A. baumannii* and blue fluorescence represents the nuclei, scale bar, 50μm. (**D**) The effects of different *A. baumannii* strains on A549 cell viability (CCK8 assay). (**E**) Evans blue dye (EB) permeability through the pulmonary epithelium of uninfected (Sham), Δ*ompA* (17978Δ*ompA*+pYMAb2) and Δ*ompA ompA*+ complemented strain-infected C57BL/6J mice measured in bronchial alveolar lavage fluid (BALF) (*n* = 3 per group). **(F)** EB-conjugated albumin (EBA, final concentration: 0.67 mg/mL) flux after infection with Δ*ompA* and Δ*ompA ompA*+ complemented strains (MOI=50). **(G)** TEER of A549 cell monolayers at multiple time points after treatment with Δ*ompA* and Δ*ompA* *ompA*+ complemented strains. (**H-K**) ELISA of IL-6 and TNF-α in mouse (*n* = 5 per group) BALF (**H and I**) and serum (**J and K**) at 48h after challenge with Δ*ompA* and Δ*ompA ompA*+ complemented strains. (**Land M**) The effects of different concentrations of C29 (**L**) and JSH-23(**M**) on A549 cell viability (CCK8 assay). (**N**) IL-1β, IL-6, IL-8, and TNF-α mRNA levels in A549 cells at 6 h after infection with different *A. baumannii* strains. Data are from three independent experiments; error bars represent standard deviation, ***P*<0.01,**P*<0.05 vs. Control (or NC, blank) group, ^##^*P*<0.01, ^#^*P*<0.05.

**FigureS2.** (**A**) A549 cell migration ability after 17978WT and 19606WT time-dependent infection was detected by using wound healing assay. (**B**) Quantification of confluence rate of A549 cells at 24 hours after infection which represent the migration ability [% wound confluence = (a − b) × 100%/a; a = Initial scratch wound area at 0 h, b = Scratch wound area at 24 h], scale bar, 200μm. (**C and D**) Localisation of E-cadherin of HBE cells infected with different *A. baumannii* strains and A549 cells transfected with pCMV-*ompA* expression plasmid was evaluated by immunofluorescence labelling. Green represents E‐cadherin and blue indicates nuclei. Scale bar: 20μm. (**E and F**) Localisation of IQGAP1 of HBE cells infected with different *A. baumannii* strains and A549 cells transfected with pCMV-*ompA* expression plasmid was evaluated by immunofluorescence labelling. Green represents IQGAP1 and blue indicates nuclei. Scale bar: 20μm. (**G**) Expression of the IQGAP1 of A549 cells transfected with pCMV-*ompA* plasmid was evaluated by western blot, IQGAP1 protein levels are expressed relative to GAPDH. (**H and I**) IQGAP1 siRNA knockdown efficiency (mRNA level and protein level) was evaluated by quantitative real-time PCR (qRT-PCR) and western blot. (**J**) Rabbit IgG was used as a negative control for co-IP assay. ***P*<0.01 vs. Control (or NC, blank) group, ^##^*P*<0.01.

**FigureS3** (**A**) The cellular localization of E-cadherin in the lung tissue of mice challenged by intratracheal injection of different *A. baumannii* strains. (n = 3 per group), scale bar, 200μm (100X), 50μm (400X). (**B**) E-cadherin cleavage fragments (arrows) detected by the western blot at 6 h after infection with different *A. baumannii* strains and inhibitor C29, cleavage fragments levels are expressed relative to full length protein (135KD). (**C**) The effects of NC siRNA on migration ability of A549 cells infected with different *A. baumannii* strains, scale bar, 200μm. (**D and E**). The growth curves of 17978WT/ △*ompA* and 19606WT /△*ompA* within 6 h in FBS free-DMEM medium and at the starting concentration of 5*10^7^ CFU/mL. (**F and G**) AUC of growth curve. (**H**) The purity of the recombinant OmpA was detected by using SDS-PAGE and Coomassie blue staining. (**I**) The mRNA expression level of inflammatory factors (IL-6, IL-8, TNF-α) under different concentrations of BSA (2.5μg/mL, 5μg/mL, 10μg/mL) treatment. (**J**) Cell migration ability under different concentrations of BSA treatment.
