# Supplementary figures and images for: Acinetobacter baumannii Outer Membrane Protein A Induces Pulmonary Epithelial Barrier Dysfunction and Bacterial Translocation Through The TLR2/IQGAP1 Axis
Source: Front Immunol. 2022 Jun 30;13:927955. doi: 10.3389/fimmu.2022.927955 (PMC9280087; doi:10.3389/fimmu.2022.927955)

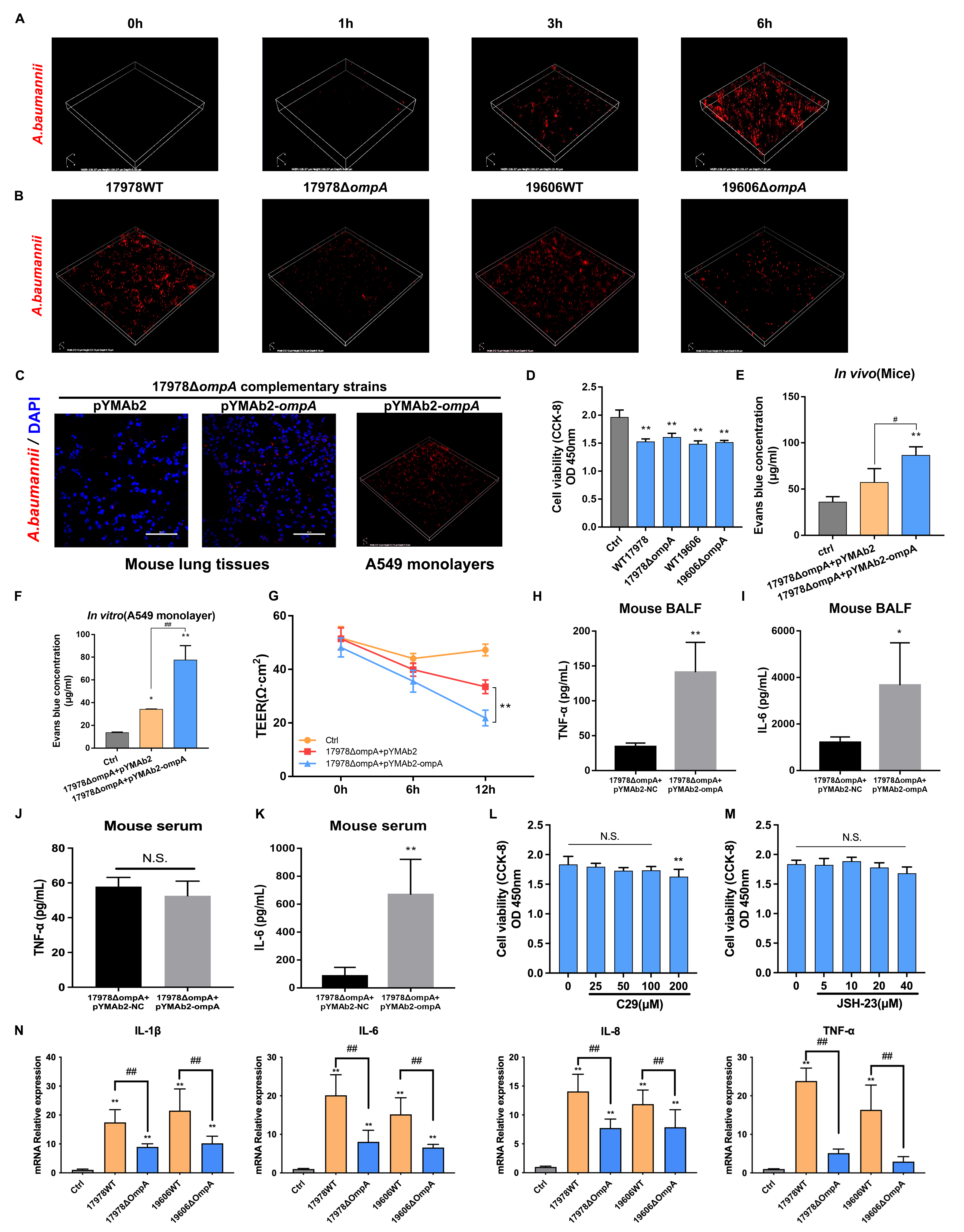

Supplement: Supplementary file 2 [file Image_1.tif]

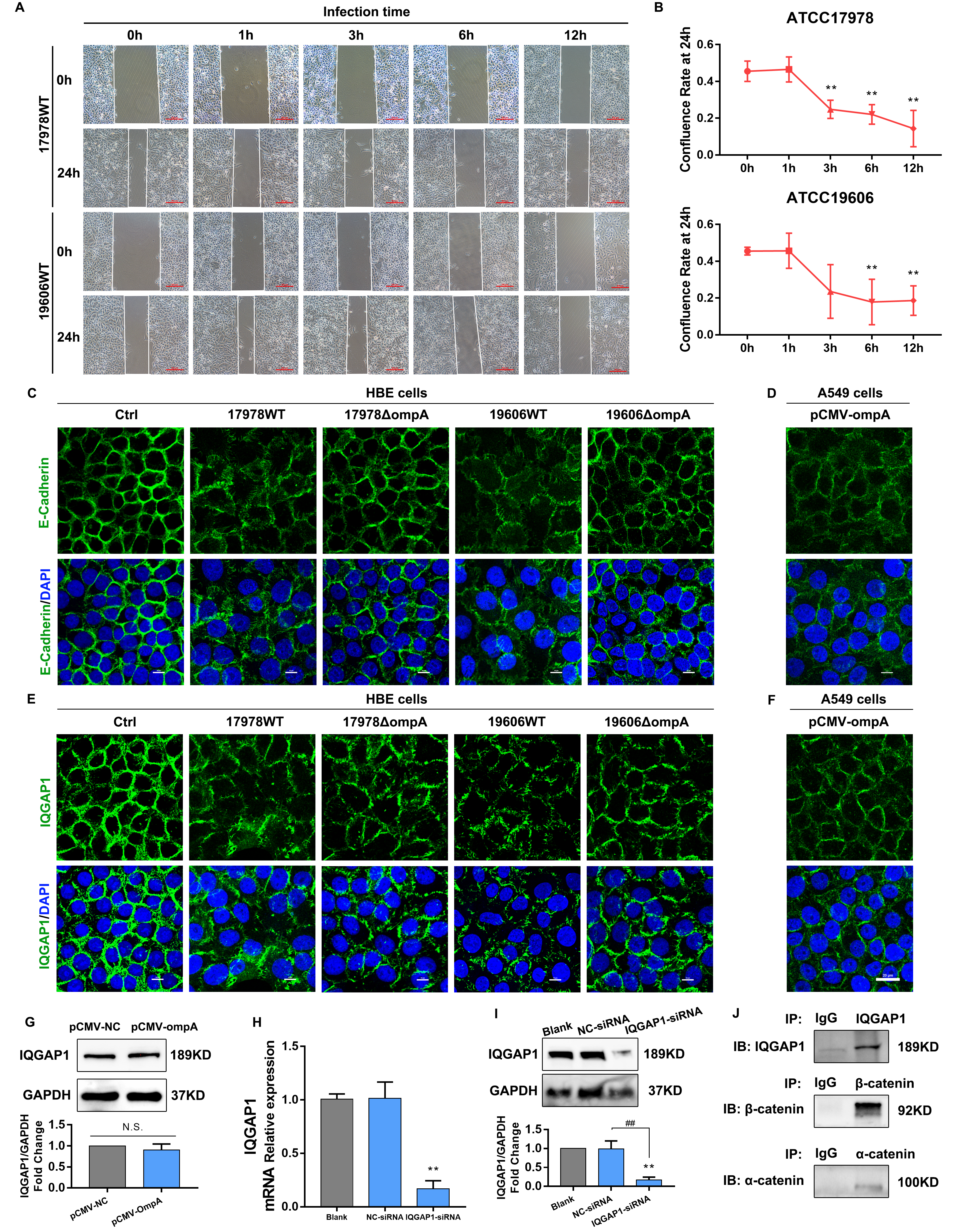

Supplement: Supplementary file 3 [file Image_2.tif]

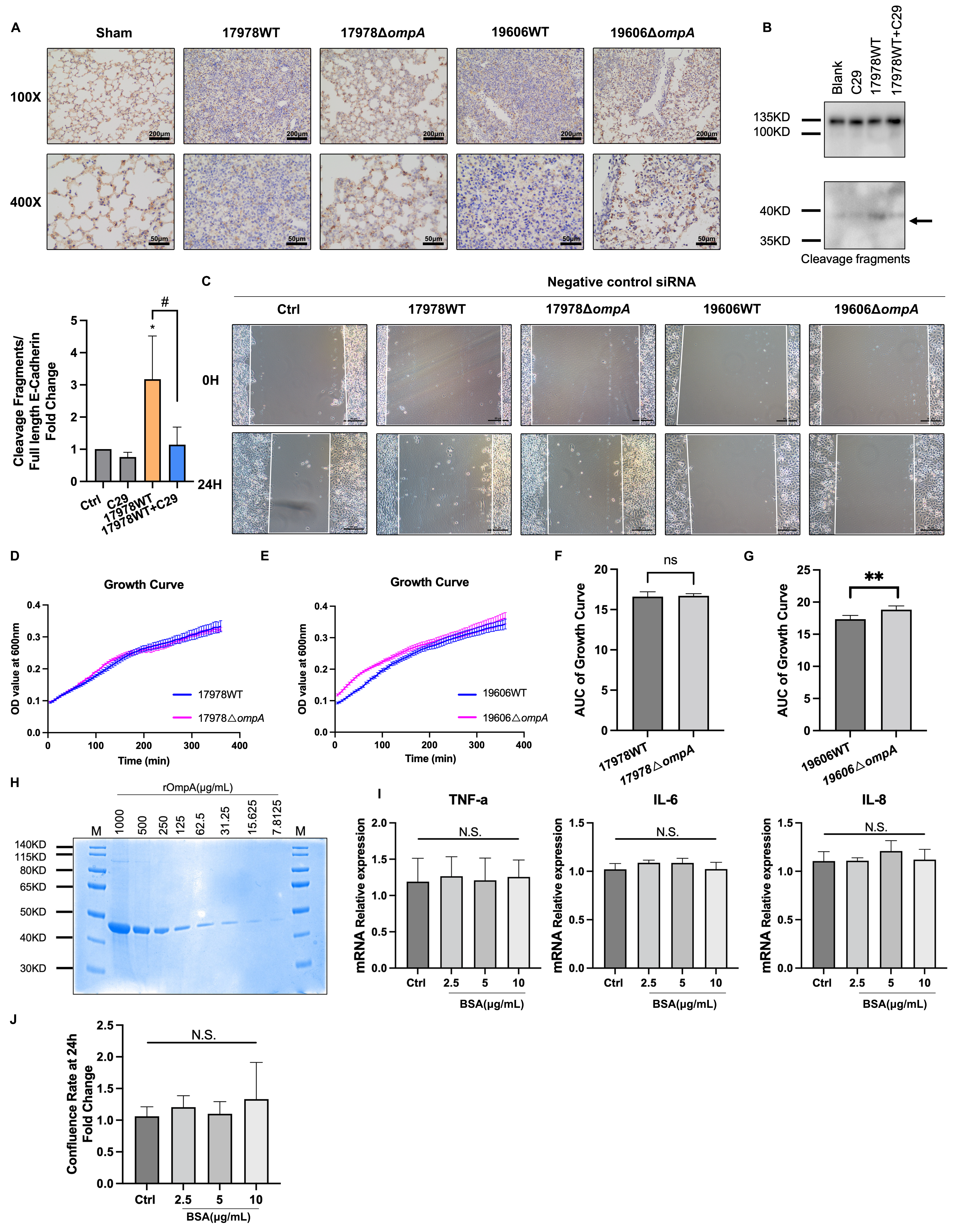

Supplement: Supplementary file 4 [file Image_3.tif]
